# Diabetes-related posttraumatic stress symptoms, resilience, and illness management among adolescents and young adults with type 1 diabetes: an exploratory study

**DOI:** 10.3389/fpsyt.2025.1615273

**Published:** 2025-07-23

**Authors:** Tamaki Hosoda-Urban, Ellen O’Donnell

**Affiliations:** ^1^ Faculty of Medicine, Tottori University, Yonago, Japan; ^2^ Department of Psychiatry, Massachusetts General Hospital, Boston, MA, United States

**Keywords:** type 1 diabetes, posttraumatic stress symptoms, adolescents and young adults, resilience, mental health, trauma, self-care, pediatric

## Abstract

**Aims:**

This study examined relationships between diabetes-related posttraumatic stress symptoms (PTSS) and depression, anxiety, resilience, Hemoglobin A1c (HbA1c), self-care behaviors, and diabetes-related distress among adolescents and young adults (AYA) with type 1 diabetes, a group navigating critical developmental transitions.

**Methods:**

Fifty AYA, aged 14–25, from a pediatric diabetes unit of an urban academic medical center participated. Diabetes-related PTSS, mental health, resilience, and diabetes self-care were assessed using validated scales. Statistical analyses examined associations and predicted likelihoods of mental health difficulties based on PTSS severity.

**Results:**

The average HbA1c was 8.36% (SD = 1.76), with 74% exceeding the recommended level. About 30% exhibited clinically relevant diabetes-related PTSS. PTSS was positively correlated with depression (r = 0.367, p = 0.009), anxiety (r = 0.435, p = 0.002), and diabetes-related distress (r = 0.436, p = 0.002), and negatively correlated with resilience (r = -0.330, p = 0.019). Higher PTSS severity increased the odds of depression (OR = 1.08, p = 0.022) and anxiety (OR = 1.09, p = 0.009), while reducing resilience (OR = 0.931, p = 0.034).

**Conclusion:**

Addressing both psychological and physical aspects of diabetes is essential. Integrating trauma-informed care and PTSS screening into routine management may improve outcomes and better support AYA during transitions. Given the study’s small sample and cross-sectional design, future longitudinal research is needed to confirm these findings.

## 1 Introduction

Adolescence and young adulthood are periods marked by developmental challenges, including navigating peer and family relationships, coping with academic pressures, meeting societal expectations, and defining career goals. For adolescents and young adults (AYA) with type 1 diabetes (T1D), these challenges are compounded by the constant demands of diabetes self-management. This includes monitoring blood glucose, administering insulin, managing diet and physical activity, and responding to symptoms such as hypoglycemia. These ongoing responsibilities can contribute to significant psychological stress, including lifestyle restrictions, fear of complications, and concerns about social stigma (1, 2).

Additionally, AYA with T1D face the anticipated future challenge of transitioning from pediatric to adult healthcare. This shift involves moving from a closely supervised, collaborative care model to one requiring greater independence and self-management. The prospect of this change can amplify existing psychological stress, especially as young patients anticipate losing long-standing supportive relationships with their pediatric diabetes care providers, often established since diagnosis (3).

In light of these stressors, AYA with T1D are more prone to mental health issues, including depression, anxiety, and distress, compared to their counterparts (1, 4, 5). Furthermore, although research is limited, a significant portion of AYA with T1D have experienced general trauma such as abuse or witnessing violence, which can lead to post-traumatic stress symptoms (PTSS) (6).

Emerging research also suggests that chronic conditions—such as severe hypoglycemic episodes, discrimination due to diabetes diagnosis, or the ongoing burden of managing the illness—can be sources of traumatic stress (6, 7). Historically, medical trauma research has centered on acute, life-threatening illnesses such as cancer or organ transplants (8, 9). This focus overlooks how chronic conditions also involve continuous traumatic stressors, which can adversely affect health outcomes.

For AYA with T1D, both diabetes-related and unrelated psychological stressors can undermine their ability to manage their condition (1, 2). Most AYA do not achieve the recommended glycemic targets set by the American Diabetes Association (10).

Resilience, defined as the capacity to adapt effectively during stress, is a psychological strength (11) particularly important for AYA with T1D. Higher resilience levels correlate with improved self-care behaviors, lower Hemoglobin A1c (HbA1c) values, fewer mental health symptoms, and higher well-being (12). However, the relationship between resilience and diabetes-related PTSS remains unexplored. Investigating diabetes-related traumatic stress is crucial, as early detection may significantly enhance psychological well-being and diabetes outcomes. Therefore, this study targets AYA diagnosed with T1D with the aim to investigate: 1) the relationships between diabetes-related PTSS and depression, anxiety, resilience, HbA1c, self-care behaviors, and diabetes-related distress; and 2) the extent to which PTSS severity predicts depression, anxiety, diabetes-related distress, resilience, and adherence to self-care.

## 2 Materials and methods

### 2.1 Study design and participants

This exploratory study was conducted in the pediatric diabetes unit of an academic medical center located in an urban area of the United States. Eligibility criteria included: 1) confirmed diagnosis of T1D; 2) age between 14–25 years; 3) English proficiency; and 4) current care at the pediatric diabetes unit. No additional exclusion criteria were applied. Participants were recruited through convenience sampling. Participant outreach was conducted through two primary channels: digital communication via the clinic’s patient portal, which included an invitation and link for registration, and direct engagement by clinic staff during routine healthcare visits.

Virtual meetings were arranged with participants to address any urgent mental health concerns (e.g., suicidality, child welfare issues), though no instances necessitated psychiatric referrals. For adolescents (14–17 years), a guardian’s presence was required for the initial consent process and to aid in filling out certain sections of the survey that inquired about health insurance details and the length of diabetes diagnosis.

Adolescents had the option to complete surveys related to mental health either independently or with their guardians present. The informed consent process and survey completion took place online, with participants sharing their screens with the research team to facilitate real-time support for any reported mental health concerns. The Mass General Brigham Institutional Review Board (IRB) granted approval for this study, ensuring all procedures adhered to established ethical standards and regulations.

A formal sample size calculation was not conducted due to the exploratory nature of the study. The final sample size was determined based on feasibility within the recruitment period.

### 2.2 Method

#### 2.2.1 Demographics

Demographic information was collected by participant self-report. Information regarding insurance and diabetes duration was collected by young adult participants (18–25 years) and adolescent participants (14–17 years) with their guardians.

#### 2.2.2 Diabetes self-care and distress

To assess diabetes self-care and health outcomes, HbA1c data were obtained from patients’ electronic medical records within 6 months of study participation, reflecting their most recent glycemic control levels. Diabetes self-care behaviors and diabetes-related distress were evaluated using self-reported, Likert-scale questionnaires. The Self-Care Inventory-Revised (SCI-R) (13), known for its strong psychometric properties, was used to assess diabetes self-care behaviors, with higher scores indicating better self-care practices. For evaluating diabetes-related distress, young adults completed the Problem Areas in Diabetes (PAID) (14), and adolescents completed the PAID-Teen (15). Both instruments have robust psychometric properties, with higher scores reflecting greater distress and emotional burnout and cutoff scores for clinically significant distress (40 for the PAID and 44 for the PAID-Teen) (14, 15).

#### 2.2.3 Mental health

Psychological symptoms were measured using self-reported, Likert-scale questionnaires. Participants were first asked to identify the most distressing diabetes-related event they experienced. Subsequently, they reported the severity of PTSS associated with this event. Young adults completed the Posttraumatic Diagnostic Scale for DSM-5 (PDS-5) (16), while adolescents completed the Child PTSD Symptom Scale 5 Self-Report (CPSS-5-SR) (17), a version of the PDS-5 adapted for younger individuals. Both scales are validated and designed to measure PTSS severity. Both the PDS-5 and CPSS-5-SR have equivalent total score ranges (0–80) and parallel severity categories (minimal, mild, moderate, severe, and very severe), enabling standardized interpretation of PTSS severity across age groups.

Depressive symptoms were assessed with the nine-item Patient Health Questionnaire (PHQ-9) for young adults (18), and the Patient Health Questionnaire for Adolescents (PHQ-A) for adolescents (19). Both versions of the PHQ are validated and widely used in clinical research. To measure anxiety symptoms, all participants completed the seven-item Generalized Anxiety Disorder Scale (GAD-7) (20), recognized for its good psychometric properties. We adopted a cutoff score of 10 or higher for both PHQ and GAD as a conventional indicator of clinically meaningful symptoms in diabetes populations (12).

#### 2.2.4 Resilience

Resilience was gauged using self-report, Likert-scale questionnaires designed to measure diabetes-related resilience. Young adults filled out the Diabetes Strength and Resilience Measure-Young Adult (DSTAR-YA) (21), and adolescents completed the DSTAR-Teen (12). Both measures employ a 5-point Likert scale to ensure compatibility across adolescent and young adult groups and are known for their robust psychometric properties. Higher scores on these measures indicate greater resilience.

### 2.3 Statistical analysis

We conducted statistical analyses using R software (version 4.3.3). PTSS scores were derived from the PDS-5 and CPSS-5-SR. Depression and anxiety scores from the PHQ-9, PHQ-A, and GAD-7 were dichotomized using established cutoffs. Diabetes self-care scores, as measured by the SCI-R, were scaled to 0-100 (13) and dichotomized at the mean. Diabetes-related distress scores, as measured by the PAID and PAID-Teen, were combined into a single categorical variable, classifying participants as ‘normal’ or ‘severely distressed’ based on standard thresholds. Resilience, assessed via DSTAR-YA and DSTAR-Teen, was merged into a single metric, averaged, and dichotomized at the mean.

To identify the strength and direction of associations between continuous PTSS scores and binary outcomes (depression, anxiety, resilience, HbA1c, diabetes self-care, diabetes-related distress), we initially conducted Point-Biserial correlations. This was supplemented by Chi-Square tests to evaluate interrelationships among the binary variables, excluding PTSS. However, when expected cell frequencies were low, we used Fisher’s Exact tests to ensure valid inference.

To assess how PTSS severity predicts other outcomes, we applied simple logistic regressions on variables flagged as significant in the Point-Biserial analyses. All statistical tests were two-tailed, with a significance threshold set at p < 0.05.

## 3 Results

### 3.1 Sample characteristics

**Table 1** summarizes sample characteristics. The sample included 50 individuals, with a mean age of 19.00 years (SD = 2.76). No missing data were reported. Most participants identified as non-Hispanic White (60.00%), were assigned female at birth (60.00%), identified as cisgender (94.00%), and had private health insurance (68.00%). The average duration of diabetes was 8.16 years (SD = 5.15).

**Table 1:** Demographics and characteristics of participants.

Variable	n (%)	Mean (SD)	Range
Age		19.00 (2.76)	14-24
Ages 14–17	14 (28.00)		
Ages 18–24	36 (72.00)		
Race/Ethnicity			
Hispanic	17 (34.00)		
Non-Hispanic White	30 (60.00)		
Non-Hispanic Black or African American	1 (2.00)		
Non-Hispanic Asian or Pacific Islander	0 (0.00)		
Non-Hispanic Biracial or multiracial	1 (2.00)		
Non-Hispanic other	1 (2.00)		
Sex assigned at birth			
Female	30 (60.00)		
Male	20 (40.00)		
Gender Identity			
Transgender and gender diverse	3 (6.00)		
Cisgender	47 (94.00)		
Insurance			
Public	16 (32.00)		
Private	34 (68.00)		
Duration of diabetes in years		8.16 (5.15)	< 1-19
A1C		8.36 (1.76)	5.10-13.00
> 7%	13 (26.00)		
7% or above	37 (74.00)		
Diabetes self-care		70.52 (12.41)	32-92
Above average	24 (48.00)		
Below average	26 (52.00)		
Diabetes-related distress			
Normal range	41 (82.00)		
Severely distressed	9 (18.00)		
Diabetes related resilience		4.17 (0.62)	1.44-4.94
Above average	28 (56.00)		
Below average	22 (44.00)		
Diabetes-related PTSS		8.80 (10.80)	
Minimal	36 (72.00)		
Mild	9 (18.00)		
Moderate	4 (8.00)		
Severe	1 (2.00)		
Very severe	0 (0.00)		
Depression		5.68 (4.77)	0-17
Non-clinical (below 10)	41 (82.00)		
Clinically relevant (10+)	9 (18.00)		
Anxiety		5.86 (5.23)	0-20
Normal (below 10)	38 (76.00)		
Clinical level (10+)	12 (24.00)		

The mean PTSS score was 8.80 (SD = 10.80), with 28.00% showing clinically relevant levels (mild or higher). Depression (PHQ-9) scores averaged 5.68 (SD = 4.77); 18.00% scored at clinical levels. Anxiety (GAD-7) averaged 5.86 (SD = 5.23), with 24.00% at a clinical level. Diabetes-related resilience (DSTAR) scores averaged 4.17 (SD = 0.62), ranging from 1.44 to 4.94.

Of the participants, 60.00% (n = 30) reported no clinically significant PTSS, depression, or anxiety symptoms. One symptom was reported by 16.00% (n = 8), two symptoms by 18.00% (n = 9), and all three symptoms by 6.00% (n = 3).

The average HbA1c was 8.36% (SD = 1.76), with 74.00% above the ADA goal of 7%. Self-care (SCI-R) scores averaged 52.89 (SD = 9.31). Diabetes distress (PAID) scores indicated that 18.00% were severely distressed.

### 3.2 Correlation between PTSS and other variables

**Table 2** presents the results of Point-Biserial correlations, Chi-Square tests, and Fisher’s Exact tests to identify the relationships among studied variables. PTSS was positively correlated with depression (r = 0.367, p = 0.009) and anxiety (r = 0.435, p = 0.002), reflecting moderate associations between PTSS and these mental health outcomes.

**Table 2:** Relationship matrix for PTSS, mental health, and diabetes management.

Variable	PTSS	Depression	Anxiety	Resilience	A1C	Diabetes self-care
	r (p-value)	OR [CI] or χ^2^ (p-value)	OR [CI] or χ^2^ (p-value)	OR [CI] or χ^2^ (p-value)	OR [CI] or χ^2^ (p-value)	OR [CI] or χ^2^ (p-value)
Depression	0.37					
	(0.009**)					
Anxiety	0.44	10.84 [1.77-86.52]				
	(0.002**)	(0.003**)				
Resilience	-0.33	0.33 [0.05-1.80]	0.66			
	(0.019*)	(0.157)	(0.416)			
A1C	0.22	3.25 [0.36-158.82]	1.05e-30	4.37		
	(0.129)	(0.414)	(1.000)	(0.037*)		
Diabetes self-care	-0.15	0.25 [0.02-1.55]	0.66	3.05	0.03	
	(0.295)	(0.142)	(0.416)	(0.081†)	(0.867)	
Diabetes-related distress	0.44	5.49 [0.81-37.50]	10.84 [1.77-86.52]	0.17 [0.015-1.05]	3.25 [0.36-158.82]	0.48 [0.07-2.64]
	(0.002**)	(0.043*)	(0.003**)	(0.032*)	(0.414)	(0.467)

r represents Point-Biserial correlation coefficients for the relationships between PTSS and other variables. OR [CI] denotes odds ratios with 95% confidence intervals, obtained from Fisher's Exact Test, used for analyzing relationships involving categorical variables with small sample sizes or low expected frequencies in contingency tables. χ² represents Chi-square statistics examining the relationships among binary variables excluding PTSS, used where expected cell counts were sufficient. Statistical significance is indicated as follows: †p < 0.10, *p < 0.05, **p < 0.01.

PTSS also showed a negative correlation with resilience (r = -0.330, p = 0.019), indicating a modest inverse relationship, and a positive correlation with diabetes-related distress (r = 0.436, p = 0.002), suggesting a moderate link between PTSS and distress related to diabetes management.

In addition to correlations with PTSS, significant associations among other variables were noted. A robust link between depression and anxiety was identified (OR = 10.84, 95% CI [1.77, 86.52], p = 0.003), indicating that participants with clinically elevated depression had more than ten times the odds of also experiencing elevated anxiety. Both depression (OR = 5.49, 95% CI [0.81, 37.50], p = 0.043) and anxiety (OR = 10.84, 95% CI [1.77, 86.52], p = 0.003) showed significant associations with diabetes-related distress, highlighting a strong connection between these psychological states and diabetes management challenges. The wide confidence interval for depression suggests variability in its impact on diabetes-related distress.

Resilience was associated with HbA1c levels (χ² = 4.37, p = 0.037) and diabetes-related distress (OR = 0.17, 95% CI [0.015, 1.05], p = 0.032), indicating that those with higher resilience were less likely to experience severe distress. However, the confidence interval reaching 1 suggests variability. The relationship between resilience and diabetes self-care showed a trend towards significance (χ² = 3.05, p = 0.081), suggesting a possible link that warrants further study.

### 3.3 Associations between PTSS severity and health-related outcomes in individuals with diabetes

**Table 3** presents results from simple logistic regressions. The findings reveal significant associations between PTSS severity and several key outcomes. For every unit increase in PTSS severity, the odds of experiencing depression increased by 8% (OR = 1.08, 95% CI [1.01–1.15], p = 0.022), and the odds of anxiety increased by 9% (OR = 1.09, 95% CI [1.02, 1.17], p = 0.009), representing small but meaningful effects. Higher PTSS severity was also linked to lower odds of resilience (OR = 0.93, 95% CI [0.87, 1.00], p = 0.034), indicating a modest inverse relationship, and greater odds of diabetes-related distress (OR = 1.10, 95% CI [1.02, 1.17], p = 0.009), suggesting a 10% increase in the likelihood of severe distress with each unit increase in PTSS severity.

**Table 3:** Association between PTSS severity and mental health, diabetes-related distress, and resilience: Logistic regression results.

Dependent variable	Odds ratio	95% CI	p-value
Depression	1.08	1.01-1.15	0.022*
Anxiety	1.09	1.02-1.17	0.009**
Resilience	0.93	0.87-0.10	0.034*
Diabetes related distress	1.10	1.02-1.17	0.009**

CI represents confidence interval. Statistical significance is indicated as follows: *p < 0.05, **p < 0.01.

## 4 Discussion

AYA with T1D face key developmental transitions, increasing their risk for both mental and physical health challenges. The goal of this study was to better understand these challenges by examining the interplay between diabetes-related PTSS and various mental and physical health indicators, with particular attention to its association with resilience. The results expand on existing literature by highlighting how PTSS stemming from diabetes-related events may be linked to well-being in this population.

Consistent with broader trends (10), a significant portion of our participants—nearly three-fourths (74%)—struggle to meet the American Diabetes Association’s HbA1c goal of below 7%. Self-care levels also align with previous findings (22). Moreover, substantial diabetes-related distress was reported by nearly one-fifth (18%) of our cohort—lower than the 30–50% typically cited in the literature (2), possibly due to differences in age, demographics, or measures used. Mental health challenges were notable in our sample. More than one-fourth exhibited mild or greater levels of diabetes-related PTSS. Depression and anxiety rates were similar to prior research (1). Overall, 40% reported at least one condition of diabetes-related PTSS, depression, or anxiety, with 24% experiencing multiple conditions.

These results indicate that AYA with T1D may face intertwined physical and mental health challenges. In particular, AYA with racial/ethnic minority or low socioeconomic status tend to exhibit compromised glycemic control and elevated HbA1c levels (23, 24) and are more vulnerable to psychiatric disorders, which is also a risk factor for compromised diabetes management (1, 23). Given our sample was predominantly White and privately insured, the findings suggest that these challenges are pervasive across AYA of diverse backgrounds. Integrated care addressing both the psychological and physical dimensions of diabetes are essential.

Diabetes-related PTSS was positively correlated with depression, anxiety, and diabetes-related distress, and negatively correlated with resilience. These findings are consistent with previous research showing that trauma-related symptoms, whether directly related to illness or not, are commonly linked to poorer mental health outcomes in individuals with diabetes (25, 26). Moreover, higher levels of diabetes-related PTSS increased the likelihood of exhibiting clinical levels of depression, anxiety, and diabetes-related distress. The observed inverse relationship between resilience and diabetes-related PTSS also aligned with a well-documented pattern (27).

Additionally, strong associations between depression, anxiety, and distress highlight the frequent co-occurrence of these conditions. These findings illustrate the complex challenges in managing both diabetes care and psychological well-being.

Our findings on diabetes-related PTSS add new insights to the literature. Comorbidity of PTSS from general trauma with mental and physical health conditions is well-documented (26, 28). However, research on PTSS from diabetes-specific events (e.g., severe hypoglycemia, diabetes-related discrimination) and their links with other mental health conditions, is extremely limited. This gap leaves the need to better understand how these conditions interrelate.

While our study design does not allow for causal conclusions, we speculate that individuals with anxiety or depression may be more likely to perceive diabetes-related stressors as traumatic compared to those without these conditions. Similarly, those with lower resilience may be more prone to view diabetes-related events as traumatic than those with higher resilience. This aligns with theories framing resilience as a protective factor in coping with adversity (29). Otherwise, individuals who have experience of diabetes-related traumatic stressors might be more likely to develop or exacerbate depressive or anxiety symptoms, as well as diabetes-related distress.

Despite the established links between depression, diabetes-related distress, and poor glycemic control (1, 2), we did not find significant associations between mental health conditions and HbA1c or self-care, possibly due to our small sample size. Given the observed associations between depression and diabetes-related PTSS, diabetes-related PTSS may have clinical relevance for diabetes management, which could be explored in future research.

Our findings underscore the need for psychological interventions tailored to the unique challenges faced by AYA with T1D. Interventions that address PTSS, depression, anxiety, and diabetes-related distress, while also strengthening resilience, can offer a holistic strategy to improve diabetes outcomes. Trauma-informed care (TIC) is a promising approach in this regard, emphasizing the importance of recognition and use of protective factors such as resilience (1, 2). TIC also considers the individual’s demographic (e.g., race, ethnicity, gender), cultural, and socioeconomic contexts, making it well-suited to address the multifaceted challenges faced by this population.

Resilience-focused, trauma-informed interventions can promote trust, safety, and collaboration with patients and families. This includes acknowledging and respecting cultural beliefs, health practices, and social contexts that shape individuals’ experiences with diabetes. Providing patient- and family-centered care that empowers youth in decision-making may enhance self-efficacy and engagement. Psychoeducation on diabetes-related stress and trauma—delivered in a culturally responsive manner—can address issues such as stigma, culturally grounded illness beliefs, and treatment expectations rooted in religious or community values. Promoting emotion regulation and coping skills—within broader support systems, including family and peers—may further strengthen resilience. Fostering a supportive environment within care teams can also reduce provider burnout and enhance care quality.

TIC also emphasizes screening for traumatic stress and minimizing re-traumatization by recognizing the potential trauma of diabetes-related experiences (30). Given the known impact of depression and anxiety on diabetes management, the American Diabetes Association advises regular screening for these conditions in diabetes care (31). However, trauma or PTSS screening is not widely implemented. In our predominantly White and privately insured sample, a notable portion nonetheless reported diabetes-related PTSS. These findings suggest that PTSS screening may be relevant across a range of socioeconomic backgrounds and highlight the value of incorporating TIC practices into standard diabetes care. Adding TIC and PTSS screening to routine care is a crucial step that may fill a key gap in the literature and improve support for AYA during transitions.

Our relatively small sample may limit the generalizability of findings and reduce power to detect significant effects. Additionally, the sample was predominantly non-Hispanic White and privately insured, which may limit the applicability of findings to more diverse populations, including racial/ethnic minorities and individuals with lower socioeconomic status. The cross-sectional design also prevents causal interpretations between PTSS, psychological factors, and diabetes outcomes. While we included HbA1c as an objective measure, reliance on self-reported data for other variables may introduce the potential for response bias. We also did not collect detailed information on concurrent medications, psychotherapy, or other interventions participants may have been receiving. These unmeasured factors may have influenced psychological outcomes and diabetes-related behaviors.

Future research should include larger and more diverse samples to improve generalizability. Longitudinal designs can clarify causal links between diabetes-related PTSS and outcomes such as glycemic control and self-care. Investigating resilience as a potential moderator and comparing diabetes-related PTSS or non-medical trauma may help inform more targeted approaches to screening and intervention.

In conclusion, our findings reveal complex and clinically relevant patterns linking diabetes-related PTSS with other psychological challenges and reduced resilience in AYA with T1D. These interrelationships suggest that individuals experiencing PTSS may be caught in a spiral of emotional distress that undermines their capacity to manage the daily demands of diabetes. Moreover, low resilience may heighten susceptibility to perceiving diabetes-related events as traumatic, reinforcing this spiral. Recognizing and addressing these dynamics is critical to designing interventions that not only reduce psychological burden but also support long-term diabetes management. Our findings underscore the importance of incorporating trauma-informed, resilience-enhancing strategies into care models for this population.

## Data Availability

The datasets presented in this article are not readily available due to restrictions imposed by the Mass General Brigham Institutional Review Board (IRB). Requests to access the datasets should be directed to Tamaki Hosoda-Urban, t.hosoda.urban@tottori-u.ac.jp.
